# Influence of Pollen Transport Dynamics on Sire Profiles and Multiple Paternity in Flowering Plants

**DOI:** 10.1371/journal.pone.0076312

**Published:** 2013-10-03

**Authors:** Randall J. Mitchell, William G. Wilson, Karsten G. Holmquist, Jeffrey D. Karron

**Affiliations:** 1 Department of Biology, University of Akron, Akron, Ohio, United States of America; 2 Department of Biology, Duke University, Durham, North Carolina, United States of America; 3 Department of Biological Sciences, University of Wisconsin-Milwaukee, Milwaukee, Wisconsin, United States of America; University of Kent, United Kingdom

## Abstract

In many flowering plants individual fruits contain a mixture of half- and full- siblings, reflecting pollination by several fathers. To better understand the mechanisms generating multiple paternity within fruits we present a theoretical framework linking pollen carryover with patterns of pollinator movement. This ‘sire profile’ model predicts that species with more extensive pollen carryover will have a greater number of mates. It also predicts that flowers on large displays, which are often probed consecutively during a single pollinator visitation sequence, will have a lower effective number of mates. We compared these predictions with observed values for bumble bee-pollinated *Mimulus ringens*, which has restricted carryover, and hummingbird-pollinated *Ipomopsis aggregata*, which has extensive carryover. The model correctly predicted that the effective number of mates is much higher in the species with more extensive carryover. This work extends our knowledge of plant mating systems by highlighting mechanisms influencing the genetic composition of sibships.

## Introduction

Multiseeded angiosperm fruits frequently contain a mixture of half- and full-siblings [1], [2]–[4] indicating that pollen from multiple donors was deposited onto the stigma and had the opportunity to fertilize ovules. Since the number of sires per fruit influences the genetic relatedness and genetic variance among siblings [5]–[7] and may have significant consequences for competitive interactions among developing seeds within fruits and among seedlings in the field [8]–[12], understanding the causes of multiple paternity can provide valuable insights on an important aspect of plant mating systems [13].

The extent of multiple paternity within fruits may be influenced by several factors, including the dynamics of pollen removal and deposition [14], [15] and patterns of pollinator movement within and among plants [2], [16]. In order to more fully explore how these factors influence effective mate number within fruits, we present a theoretical framework which links models of pollen carryover to patterns of paternity. Using these models we address the following questions: (1) How does pollen carryover influence the number and diversity of pollen donors siring seeds within fruits? (2) How do within-plant pollinator movements influence the number and diversity of pollen donors siring seeds within fruits? After presenting the theoretical framework, we test the models with empirical data from two species that differ markedly in the extent of pollen carryover.

## Results

### Modeling the Sire Profile for Recipient Fruits

Pollen carryover from a donor flower can be modeled using a variety of different formulations, the simplest useful version being an exponential decay [17]. Although more-complicated models (e.g., two-compartment) that account for features such as pollen layering tend to fit observed patterns more closely [17]–[19], here we use the slightly simpler carryover dynamics of the single exponential to facilitate the addition of new ecological complexities in an analytically approachable manner. In the single exponential decay model [17] the amount of pollen from the donor flower that arrives on the k^th^ flower visited (C_k_) is a function of the amount of pollen carried away by the pollinator (A_o_), and the proportion of pollen removed from the pollinator by the stigmas of flowers probed sequentially by the pollinator (ρ; see Table 1 for a summary of parameter symbols and definitions):
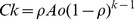



**Table 1:** Summary of parameters used in the text.

Symbol	Definition
C_k_	The amount of pollen from the donor flower that arrives on the k^th^ flower visited
A_o_	The amount of pollen carried away from a flower by the pollinator
ρ	Pollen carryover parameter; the proportion of pollen removed from the pollinator by the stigma of each visited flower
τ	The probability of departing a plant after visiting a flower
γ	Product of the probability of remaining at a plant after visiting a flower, and the proportion of pollen not deposited on a flower by the pollinator ( = (1- τ)(1- ρ)).

Because this modeling approach focuses on the destination of pollen exported from a single flower, it can be termed a “donor-centered” view of pollen transfer. However, studies of mating patterns in plants more commonly focus on the success of one or more pollen donors in siring seeds of a single flower [2], [15]. This is a “recipient-centered” view of pollen dispersal in that it provides a profile of the pollen arriving at a flower, and therefore of the sires likely to be represented in a fruit. In the past, technological limits on the number of donors that could be identified prevented detailed consideration of sire profiles. However, recent advances [3], [13], [20] have made paternity assignment for larger numbers of sires feasible, increasing the need for such models.

Rephrasing the exponential decay model above can give a recipient-centered view (sire profile) by predicting the proportion of pollen arriving on a recipient’s stigma from each of the prior donors as the result of a single pollinator visit [14], [21].

**Figure 1:**
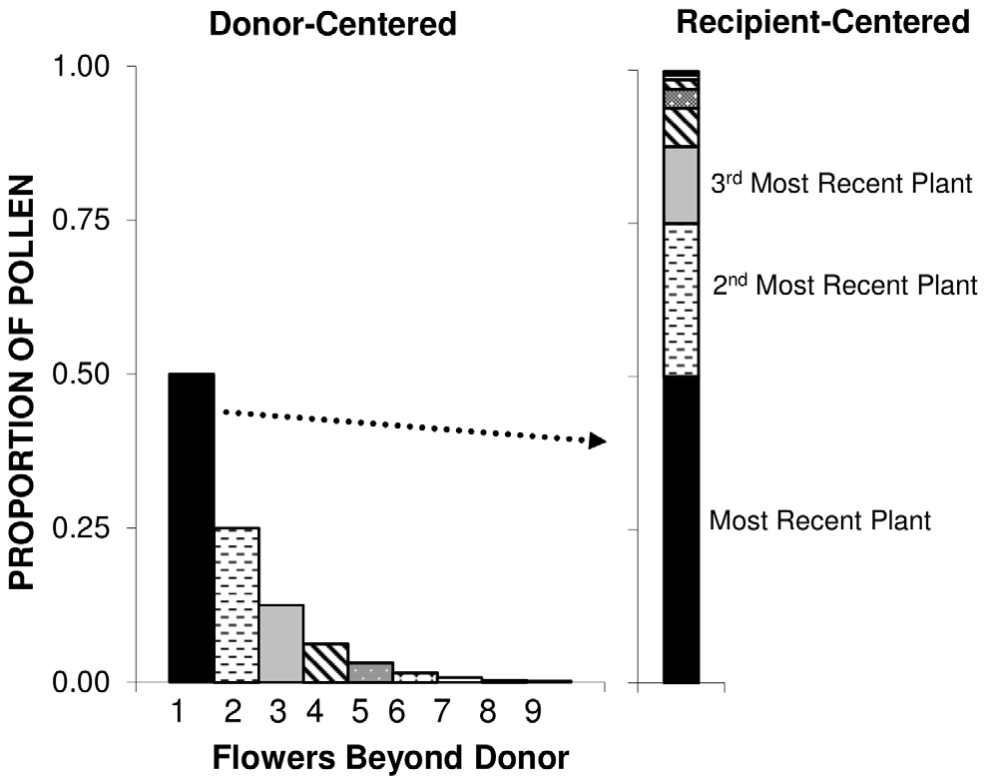
Sire profile construction. The sire profile for a single recipient flower is formed by summing the pollen contributions of previously visited flowers. In this example each plant has a single flower. Values are from the exponential carryover equation (C_k_) with ρ = 0.5.

Assuming that A_o_ and ρ are the same for all flowers, a single pollinator visit should deposit a fraction of pollen (ρ(1-ρ)^0^) from the previous flower, a fraction (ρ(1-ρ)^1^) from the 2^nd^ preceding flower, and so forth 
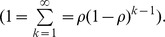
 If pollinators visit only one flower on each plant during a foraging sequence, this equation (which we will refer to as the “*C_k_* model”) predicts the mix of pollen arriving on the focal flower (Fig. 1). The recipient-centered sire profile for a single fruit is the direct outcome of the individual pollen dispersal curves for each of the previously-visited donor flowers. Assuming that pollen from different donors is equally able to sire seeds, this also predicts patterns of paternity within fruits following single pollinator visits.

#### Multiple flowers on a plant

**Figure 2:**
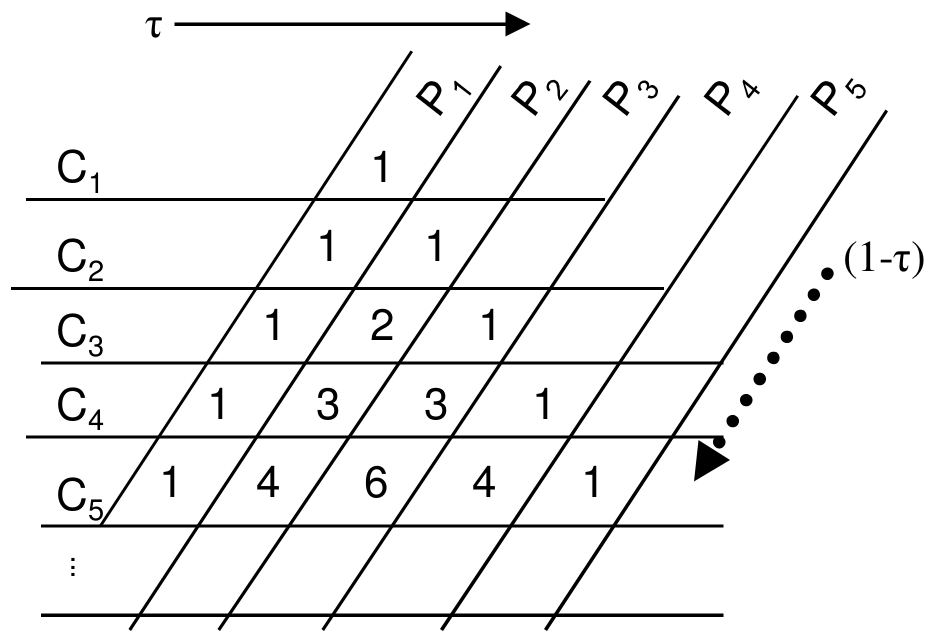
Binomial coefficients used in generating the P_i_ model. Plants are on diagonals, flowers are on rows.



Since animals often visit more than one flower on a plant, it is important to account for within-plant (geitonogamous) pollinator movements on multi-flowered plants. When pollinators visit more than one flower on a plant, the amount of pollen from a plant that is loaded on the pollinator will vary with the number of flowers visited. The effects of this sort of pollinator foraging on patterns of mating can be modeled by assuming that pollinators exhibit a constant probability of departing a plant, τ [22], [23]. Assume that τ is constant in a plant population (for example, all plants might have similar floral display sizes). Given constant τ, the amount of pollen exported from Plant 1 can be characterized by the dispersal from Flower 1 (C_1_), plus the dispersal from a second flower (C_2_) times the probability that the pollinator remains at plant 1 to visit that second flower (1- τ), and so forth. Therefore, pollen receipt from Plant 1 (P_1_) can be characterized by:
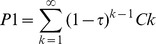



The same approach applies to other plants in the foraging sequence. In general, the number of possible ways that the *i* ^th^ previous plant can contribute pollen as the *k* ^th^ donor is represented by Pascal’s triangle, in which flowers are the horizontal elements, and plants are the diagonal elements (Fig. 2). For example, the ‘3’ situated at C4,P3 means that there are three ways for the third prior plant to contribute the fourth donor visit: there is one and only one repeated visit at any of the three prior plants. Each of these possible visitation pathways must be counted separately, hence, the Pascal’s triangle formulation. The expected contribution of pollen from the *i* ^th^ plant to a recipient flower is a diagonal sum along the triangle of the number of possible ways that pollen might be carried over from a particular donor in the sequence. Put another way, with flowers indexed by *k* and plants indexed by *i*, the relationship can be summarized as.

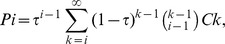
where the binomial coefficient indicates the many possible combinations of geitonogamous and outcross visits that are possible for long visitation bouts (Fig. 2). This will henceforth be referred to as the *P_i_* model of pollen carryover. The *P_i_* model can be used when plants have multiple open flowers to determine the sire profile of the first flower visited on a plant.

As an example, we can consider exponential pollen carryover, and replace C_k_ with the pollen carryover curve C_k_ = ρA_o_(1-ρ)^k-1^. Then,
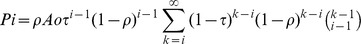



Defining for simplicity γ = (1- τ)(1- ρ), we obtain
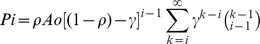
in which we must be clear to define 0^0^ = 1 and 0^n>0^ = 0. Note that the case τ = 1 collapses to the expected result for exponential pollen carryover without the potential for within-plant movements [21].

The above expression for P*_i_* applies when the focal recipient flower is the first flower visited on a plant, and it does not allow for the possibility that the focal flower receives geitonogamous self pollen from immediately prior visits to other flowers on the same plant. In other words, we explicitly assume that P_0_ = 0, where the index *i* = 0 represents the recipient flower’s plant. However, within-plant movements can be accounted for by recasting the previous plant visited as the probability (1-τ) that the pollinator did not switch plants. This accounting leads to the expression,
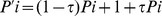



This expression models deposition of both outcross and geitonogamous self pollen onto the stigma of a focal flower. Therefore we will refer to it as the *P′_i_* model. The *P′_i_* model applies when plants have multiple open flower, and is used to determine the sire profile when geitonogamous selfing is possible for the recipient flower.

The models described above generate predictions about the composition of pollen loads arriving on stigmas. However, in empirical studies it is often desirable or necessary to determine the sire profile from seedling genotypes. Because genotype samples seldom include more than 10–20 offspring per fruit, the realized sire profile will be strongly influenced by sampling variance. Therefore, to evaluate how sire profiles change among models, and as a function of model parameters, we used a SAS macro to randomly sample a given number (usually 20) of ‘genotyped offspring’ from the pollen profile generated by the model. To do this we assumed that paternity is determined solely by the proportional representation of pollen in the pollen load – that is, that there is no post-pollination screening or differential pollen tube growth. We then sampled a given number of seeds from this distribution for a large number (usually 1000 or 10,000) of simulated fruits, and then calculated mean mate diversity.

We used as our primary estimate of mate diversity the correlation of outcrossed paternity within fruits, r_p_, which is the proportion of full sibs among outcrossed sibs [6]. An important advantage of r_p_ over alternative measures of mate diversity (such as number of sires) is that r_p_ is unaffected by the number of seeds sampled [24]. The inverse of r_p_ is an index of the ‘effective number of mates’, the number of individuals contributing to a random mating pool that would generate the observed r_p_ [24]. For comparison, we also calculated other common estimators of mate diversity within a fruit, including the number of outcross sires, the total number of sires (including selfs), and the Shannon-Weaver index of diversity.

### Model Predictions

**Figure 3:**
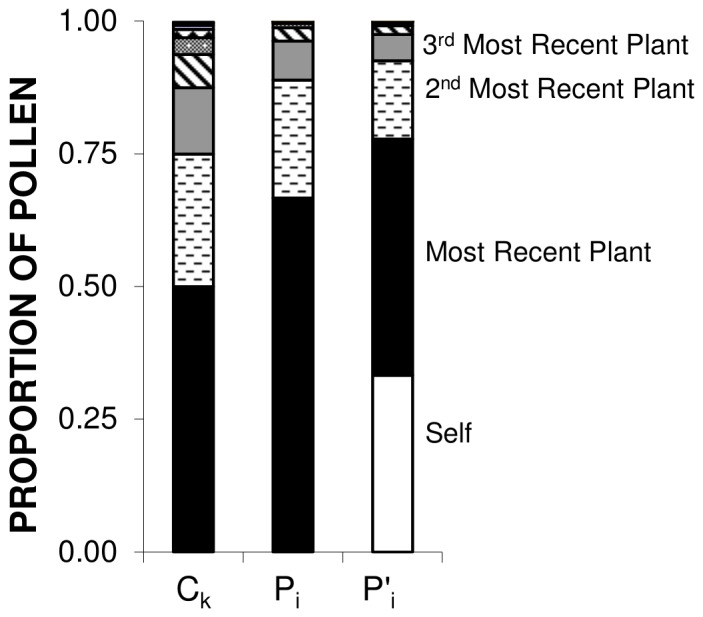
Sire profiles for the three models. The Ck model assumes that every plant has a single flower. The P_i_ model assumes that plants have multiple flowers, but that the focal flower is the first flower probed on a plant, and doesn’t receive geitonogamous self-pollen. The P′_i_ model assumes deposition of both outcross and geitonogamous self-pollen onto the stigma of a focal flower. In this example ρ = 0.5, and τ = 0.5.

Fig. 3 shows representative sire profiles for these three models. The three models (C_k,_ P_i_, P′_i_) allow progressively more within plant movement and variation in visitor itinerary, and generate progressively less diversity in the outcross sire profile. As expected, the C_k_ model (in which each flower is on a different plant) generates the greatest diversity of mates by all measures (Table 2). Likewise, the P_i_ and P′_i_ models give very similar results, but the latter, which allows geitonogamous self-pollen deposition onto the stigma of the focal flower, (with probability τ) has slightly less diversity of mates than the P_i_ model, which does not allow selfing on the first flower. Note that the possibility of within plant movement allowed in the P_i_ and P′_i_ models decreases the number of sires that might be detected because a large fraction of sampled offspring are selfs, and therefore not part of outcross mate diversity., and because visiting several flowers on prior plants increases the representation of the recent donors relative to those earlier in the visitation sequence.

**Table 2:** Measures of mate diversity for the three sire profile models.*

Model	C_k_: Single flowered plants	P_i_: 1^st^ flower on a multi-flowered plant	P′_i_- Multi-flowered plants
r_p_ = correlation of outcrossed paternity	0.334±0.001	0.499±0.001	0.502±0.001
1/r_p_ = effective number of outcross mates	3.185±0.008	2.103±0.005	2.154±0.006
Sires/fruit (excluding Selfs)	4.680±0.010	3.408±0.008	3.042±0.001
Sires/fruit (including Selfs)	4.680±0.010	3.408±0.008	4.042±0.001
Shannon index for Outcrossed seeds	1.211±0.002	0.845±0.001	0.797±0.002

* Models estimated using ρ = 0.5, and τ = 0.5. Values shown are mean ± SE for N = 10,000 replicate fruits, each fruit with 20 simulated seeds. Mean 1/r_p_ is not equal to 1/mean r_p_ because the values are calculated separately for each fruit.

To explore the influence of ρ and τ on mating patterns, we used two approaches to examine the P′*_i_* model in more detail. First, we looked at how the proportional representation of sires varies as a function of varying ρ and τ separately. In both cases we took ρ = τ = 0.5 as a starting point (these are reasonable values for many bee-pollinated plants; for ρ see [26] and for τ see refs [16], [19], [22]). We then altered these factors one at a time, and presented the resulting sire profile.

**Figure 4:**
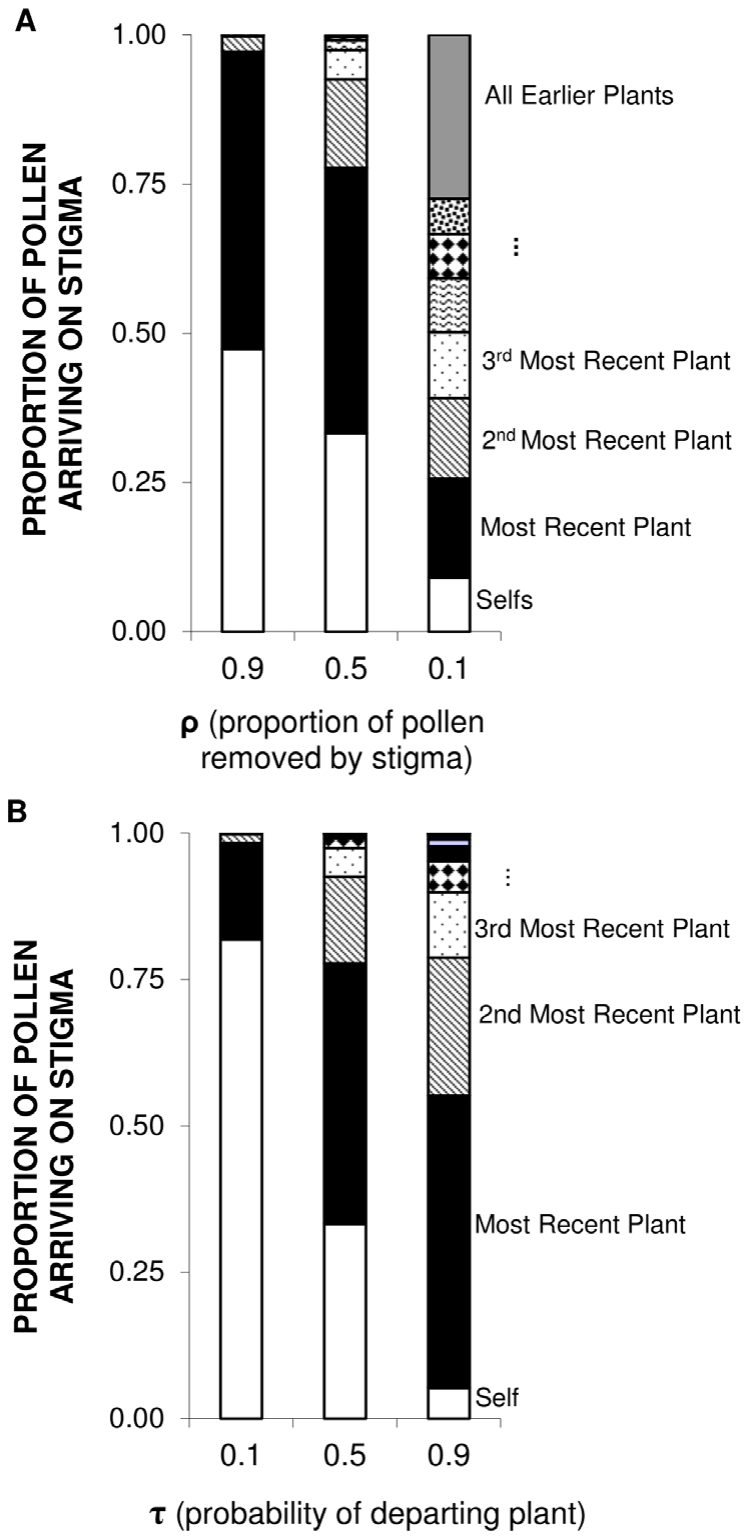
Effects of varying carryover fraction (ρ) and probability of departing a plant (τ). Sire profiles (proportion of pollen arriving on a flower’s stigma from previously visited plants during a single floral probe) are derived using the P′_i_ model. A) Effect of varying ρ (τ kept constant at 0.5). Shading distinguishes the donors; for ρ = 0.1, the first 15 donors are indicated by distinct patterns, while the graded gray shading for the top bar denotes the total for plants earlier in the visitation sequence, each of which represents less than 0.5% of the seeds sired on that flower. B) Effect of varying τ (ρ kept constant at 0.5).

Keeping τ constant, as ρ decreases (reducing the proportion of pollen removed by the stigma, and therefore reflecting increased pollen carryover), the number of outcross donors, their diversity, and evenness of donors increases (Fig. 4). Indeed, for very small values of ρ the number of potential donors becomes extremely large, so that even very distant plants may be represented in the pollen load deposited on a particular stigma. Extensive carryover (small values of ρ) also reduces the contribution of geitonogamy to the sire profile.

A similar but less dramatic response occurs for changes in τ, keeping ρ constant. As τ increases (pollinators move more often between plants), the number of donors represented in the pollen load increases (Fig. 4), as does the diversity and evenness of representation of donors. Note that τ = 1 corresponds to the C_k_ model, where within plant movements do not occur. This supports the intuition that fewer movements between plants should strongly reduce mate diversity. However, the influence of τ across nearly the full range of possible values from 0 to 1 is less than that of ρ across its full range of 0 to 1, suggesting that pollen carryover properties have more noticeable effects on sire profiles than do within-plant movements.

**Figure 5:**
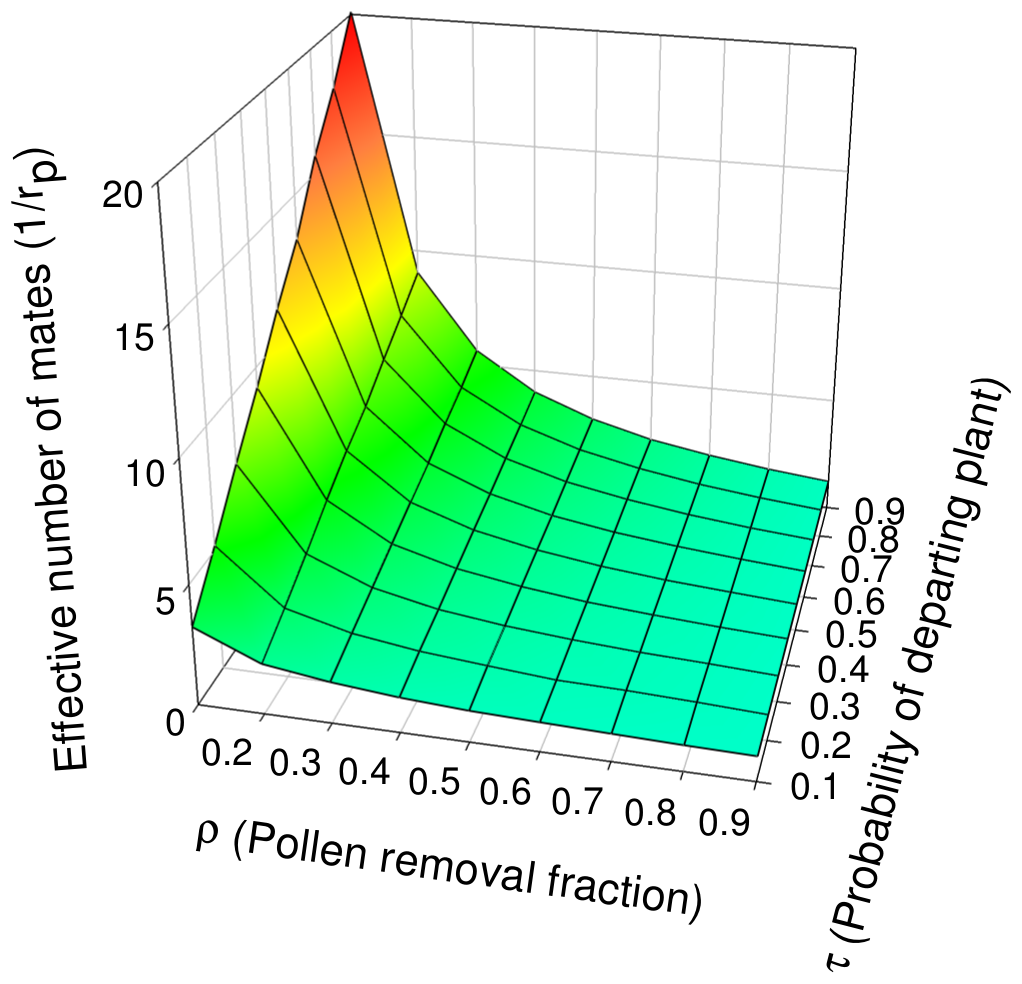
Effects of ρ and τ on mate diversity in the P′_i_ model. Mate diversity is indicated by the effective number of mates (1/r_p_). Values shown are means from 1000 simulated fruits of 20 seeds.

Our second approach to investigating the effect of varying pollen carryover and pollinator movements (ρ and τ) was to evaluate how the effective number of mates (1/r_p_) varies across combinations of values for those parameters (Fig. 5). We again found that mate diversity is greatest when ρ is small (there is extensive pollen carryover) and τ is large (pollinators move frequently between plants). These effects are fairly additive, so that the effects of τ and ρ together on diversity of mates are mutually consistent. However there are some synergies; when pollen carryover is restricted, behavior has a less noticeable effect on mate diversity. In contrast, when carryover is extensive, behavior can have stronger effects. Note that the converse also appears to be true; if pollinators rarely move between plants, increased carryover (decreasing ρ) has little effect, since most of the pollen carryover occurs during sequential geitonogamous moves on a large display. In general, the effect of τ on mate diversity is fairly linear, but when ρ is below ∼0.3 the response is curvilinear. Likewise, when pollinators rarely move between plants, increased carryover (decreasing ρ) has little effect, since most of the pollen is deposited on the same plant between the many sequential geitonogamous moves on a large display.

### Comparing Model Predictions to Observed Results

To compare the predictions of this model to observed values requires empirical estimates of the number of sires per fruit(1/r_p_), pollen carryover (ρ), and pollinator movement probabilities (τ). We are aware of only two datasets satisfying all of these requirements.

The first dataset comes from our work with *Mimulus ringens* L (Phrymaceae) [15], in which we scored mate diversity for a number of flowers that received a single probe from a freely foraging bumble bee, and genotyped 20 seeds per fruit to estimate sire profiles. These empirical estimates of mate diversity (including values of 1/r_p_ not previously reported) can be compared to the model’s predictions. To generate those predictions we used empirical estimates of τ and ρ. The probability of departing a plant (τ) can be estimated as the fraction of all plant visits during which the bee left after probing one flower. For our 2006 [15] study τ was 0.492 (J.D. Karron and R.J. Mitchell, unpublished data). Since all floral displays in this study were trimmed to four open flowers, τ was the same for all of them. In separate work we estimated ρ to be 0.68 [19], [25]. Note that this estimate of ρ is among the shortest reported pollen carryover curves [26], [27], and therefore our estimates for *M. ringens* provide a look at one extreme of the range of possible values.

For a multi-flowered self-compatible plant such as *M. ringens* the P′*_i_* model is most appropriate. We found that the observed and expected values for number of sires (out of 20 seeds/fruit) were very similar (observed: 3.12±0.44 sires; N = 17; expected: 3.23±0.15; mean expectation is based on 1000 replicate fruits, with SE based on observed N of 17). This agreement supports the idea that the model captures some of the important features of the pollination process. In contrast, the effective number of sires (1/r_p_) was far greater for the observed data (3.97±0.60, N = 17), than was predicted by the model (1.56±0.23; based on 1000 replicate fruits, SE based on N = 17). This disagreement indicates that the representation of mates in the observed data is more equitable than predicted by the model, so that there is little numerical dominance of one donor over the others. Possible explanations for the mismatch between observed and expected values for 1/r_p_ include: (1) Postpollination processes that tend to equalize sire representation [7], [10], [28], or favor some outcross donors over others. (2) Pollen dispersal curves that show greater gene movement than is predicted by the single exponential model (C_k_) used in this study; future modeling efforts should investigate this possibility by incorporating a double exponential or two-compartment pollen carryover function [17], [18].

The second dataset available to test our model comes from hummingbird-pollinated *Ipomopsis aggregata* (Pursh) V.E. Grant (Polemoniaceae). Campbell [16] reported extensive multiple paternity in this species, with a mean of 4.4 sires per fruit in samples averaging ∼7 seeds/multiseeded-fruit. For this self-incompatible species the P*_i_* model is most appropriate for generating expected values, since that model does not allow geitonogamous selfing of the focal flower but does involve multi-flowered plants. Campbell [16] estimated τ at 0.5, similar to the value for *M. ringens,* and Waser [29] estimated ρ for *I. aggregata* to be 0.033, among the lowest values yet reported [26], [27], and indicating extensive pollen carryover. This serves as a useful contrast to the very restricted level of pollen carryover for *M. ringens* (see above). Flowers of *I. aggregata* typically receive one pollinator probe during female phase [16]. When these parameter values are used in the P*_i_* model, sampling 7 seeds per fruit (near the average for Campbell’s study), the model predicts even more sires per fruit than were observed (N sires out of 7 seeds = 6.09±0.03). Remarkably, the expected value for the effective number of mates is much greater than the number of seeds typically produced by this plant (seeds per fruit is typically 5–10, while 1/r_p_ = ±17.11±0.23). Campbell did not report the effective number of mates for that study, so we cannot directly compare observed and expected values, although Campbell [16] observed that nearly every seed had a different father, as the model predicts.

The two examples above probably bracket most of the variation in pollen carryover and resulting sire profiles that are likely to be observed in nature: *M. ringens* represents very restricted pollen carryover, and *I. aggregata* represents extensive carryover. As expected based on ρ, the observed number of mates in *M. ringens* is much less than that observed in *I. aggregata*, supporting the basic conclusion of the model, that more extensive pollen carryover should generate greater mate diversity.

## Discussion

These models demonstrate that extensive pollen carryover is likely to increase the diversity and number of pollen donors siring seeds following individual pollinator probes. They also indicate that the effect of pollen carryover on sire profiles is much more noticeable than the effect of geitonogamous pollinator movements. This suggests that quantification of pollen carryover is especially important for a mechanistic understanding of the determinants of mate diversity. Studies that document how different pollinator species influence sire profiles for a given plant species are likely to be especially informative.

Although less pronounced than the effect of carryover, geitonogamy caused by long pollinator tenure on many-flowered plants also influences mate diversity within fruits. This suggests the potential for a tradeoff between pollinator attraction and the opportunity for plants to increase genetic diversity of progeny within fruits. Plants with many flowers may be more attractive to pollinators, but the long tenures of visitors on such plants may encourage geitonogamous selfing, pollen discounting, and reduced diversity of mates within fruits [23], [30]. However, in prior work with *M. ringens* we did not detect any relationship between floral display and levels of multiple paternity within fruits [2]. This may reflect the small effect size expected for changes in floral display; in that experiment, τ ranged from 0.795 for 2 flowered plants (giving an expected value of 1/r_p_ of 1.83) to τ = 0.303 for 16 flowered plants (expected value of 1/r_p_ = 1.39). The rather small difference in mate diversity caused by such a large change in patterns of pollinator movement suggests that very large sample sizes will be required to document the small expected effect. Other factors, such as multiple probes to flowers by separate pollinators (see below), may also reduce the effect of display on mate diversity.

Our results highlight the possibility, and even the likelihood, of uneven representation of sires in pollen loads and individual fruits. When sires differ greatly in their success at siring the seeds in a fruit, simply counting the number of mates does not adequately summarize paternal diversity (especially because the number of sires varies with the number of seeds sampled). Therefore it is important to report measures that account for or indicate unevenness of representation and the number of seeds sampled, including 1/r_p_ [6] and other indices [31]. Unequal representation of sires, even in single-probed flowers, may occur for many reasons beyond the pollen carryover and pollinator movement patterns we considered. For example, other factors that may influence the evenness of sires within fruits include clumping of pollen (e.g., pollinia, viscin threads [32], the extent of pollinator grooming, post-pollination screening, clonal structure of the plant population, and the spatial separation of plants and flowers. Therefore, we anticipate that unevenness within fruits should be the rule rather than the exception. Note that our models predict that disparities in donor representation should be highest when pollen carryover is short (Fig. 4a), suggesting that comparisons of, for instance, sire profiles for bumble bee vs. hummingbird visits to a single species, or of related plants that are visited by different taxa might be especially interesting [33], [34].

A necessary simplification required for this model is that we only considered flowers probed a single time. In some species flowers may receive pollen from several distinct probes that are separated in time [15]. Consideration of how this might affect mate diversity is considerably more complicated than what we modeled here. Such a model would need to account for many factors, including: (1) the number of probes received by flowers, (2) the time separating probes and how this affects the timing of arrival of pollen tubes at ovules, (3) the relative amounts of pollen deposited by each probe, (4) the foraging itinerary of the pollinators (which determines the similarity in identity and abundance of different sires brought in by different probes), (5) the extent of within-flower selfing (which may be different for the first and later probes to a flower), (6) variation among probes in the amount of geitonogamous self-pollination [35], (7) pollen priority effects that may provide an advantage [36] to earlier arriving pollen, and (8) temporal variation in postpollination screening among pollen tubes. Detailed examination of the effect of multiple probes on paternity is beyond the scope of this paper, although it is likely that mate diversity will increase with number of probes [15]. Other areas for improvement of the models would be to incorporate features such as prior and facilitated self-pollination, negative effects of self pollen in self-incompatible species, and effects of interspecific pollinator movements on the extent of pollen carryover.

Our results also suggest that mate diversity will increase when pollen carryover is extensive. This implies that the effect of different pollinator taxa on a plant’s mating success may reflect not only pollinator abundance and per-visit pollen deposition [37], [38], but also the identity of the pollen delivered by those pollinators (see [39]). Bees and other pollen-harvesting (or intensively grooming) visitors are likely to have high values of ρ (less extensive pollen carryover) compared to pollinators such as hummingbirds and hawkmoths [26], [27], and often differ in the amount of pollen deposited per visit [27], [40]. This raises the possibility that plants may at times face an important but largely unexplored tradeoff between the quantity of pollen and the diversity of mates delivered by visits from different pollinators (see [41], [42]). Likewise, factors that influence pollen carryover, such as pollinator sharing and competition for pollination, may affect selfing rates and mate diversity as well as seed production and other more commonly studied quantitative aspects of reproduction [33], [43].

Our findings demonstrate that the dynamics of pollen carryover and patterns of pollinator foraging are likely to influence two important aspects of plant mating systems: the diversity of pollen donors siring seeds within fruits, and the proportion of seeds sired by outcross pollen donors. Few studies have quantified pollen carryover, pollinator movements, geitonogamous selfing, and patterns of multiple paternity in a single population. Additional research documenting the interplay amongst these parameters is critically needed. Some of the most informative studies will combine meticulous field experimentation with unambiguous paternity assignment, enabling researchers to quantify how single visits by different pollinator species influence the genetic composition of sibships.
